# Immunoprotectivity of HLA-A2 CTL Peptides Derived from Respiratory Syncytial Virus Fusion Protein in HLA-A2 Transgenic Mouse

**DOI:** 10.1371/journal.pone.0025500

**Published:** 2011-09-29

**Authors:** Hsiao-Yun Shao, Yi-Wen Lin, Shu-Ling Yu, Hsiang-Yin Lin, Ebenezer Chitra, Yung-Chen Chang, Charles Sia, Pele Chong, Ming-Tao Hsu, Olivia L. Wei, Yen-Hung Chow

**Affiliations:** 1 Institute of Infectious Disease and Vaccinology, National Health Research Institutes, Miaoli County, Taiwan, Republic of China; 2 Graduate Program of Biotechnology in Medicine, Institute of Molecular Medicine, National Tsing Hua University, Hsinchu, Taiwan; 3 The Graduate Division of Biological and Biomedical Sciences (GDBBS), Emory University, Atlanta, Georgia, United States of America; George Mason University, United States of America

## Abstract

Identification of HLA-restricted CD8^+^ T cell epitopes is important to study RSV-induced immunity and illness. We algorithmically analyzed the sequence of the fusion protein (F) of respiratory syncytial virus (RSV) and generated synthetic peptides that can potentially bind to HLA-A*0201. Four out of the twenty-five 9-mer peptides tested: peptides 3 (F33–41), 13 (F214–222), 14 (F273–281), and 23 (F559–567), were found to bind to HLA-A*0201 with moderate to high affinity and were capable of inducing IFN-γ and IL-2 secretion in lymphocytes from HLA-A*0201 transgenic (HLA-Tg) mice pre-immunized with RSV or recombinant adenovirus expressing RSV F. HLA-Tg mice were immunized with these four peptides and were found to induce both Th1 and CD8^+^ T cell responses in *in vitro* secondary recall. Effector responses induced by these peptides were observed to confer differential protection against live RSV challenge. These peptides also caused better recovery of body weight loss induced by RSV. A significant reduction of lung viral load was observed in mice immunized with peptide 23, which appeared to enhance the levels of inflammatory chemokines (CCL17, CCL22, and IL-18) but did not increase eosinophil infiltration in the lungs. Whereas, significant reduction of infiltrated eosinophils induced by RSV infection was found in mice pre-immunized with peptide 13. Our results suggest that HLA-A2-restricted epitopes of RSV F protein could be useful for the development of epitope-based RSV vaccine.

## Introduction

Respiratory syncytial virus (RSV) induces respiratory disease in children and the elderly around the globe [1], [2], [3], and till now, there is no effective prophylactic vaccine available against RSV infection. Previous attempts in developing a vaccine using formalin-inactivated RSV failed because it exacerbated the disease upon subsequent infection in some cases [4]. Therefore, study and development of a vaccine against RSV is a priority to global welfare. Many studies conducted in animal models have convincingly shown that not only neutralizing antibodies but also CD8^+^ cytotoxic T lymphocytes (CTLs) confer protection against viral infections. The role of CTLs seems to be dual in RSV infection. Adoptive transfer of CD8^+^ CTLs offers protection to recipient BALB/c mice from subsequent infection by limiting viral replication *in vivo* [5], and also inhibits RSV vaccine induced pulmonary eosinophilia [6]. This function is dependent on the numbers of CD8^+^ CTLs in the lungs to inhibit pulmonary eosinophilia as well as the production of type 2 helper T cell (Th2) associated chemokines in mice [7]. On the other hand, excess CD8^+^ CTLs contributed to RSV-induced lung pathogenesis in mice [5], [8]; they were detected more readily in human adults who develop mild symptoms following virus exposure [9], but not so clear in infants [6]. In addition to CD8^+^ T cells, studies of RSV infection reveal that CD4^+^ T cell concurrence leads to IL-2 secretion, restores CTL activation in RSV-infected respiratory tract [10] and increases CD8^+^ T cell activation *via* IFN-γ production after *in vitro* peptide stimulation [11]. In contrast, IL-4 secreted from CD4^+^ T cells can abrogate CD8^+^ T cell response and diminish viral clearance [12], [13].

While T cell responses are required to clear RSV from the lungs, studies in murine model show that CD8^+^ T cell response against the fusion protein (F) regulates the outcome of CD4^+^ T cell responses in preventing vaccine-enhanced disease [14]. RSV challenge of BALB/c mice previously immunized with vaccinia virus (vacv) expressing RSV F protein does not lead to the development of pulmonary eosinophilia [14], [15], [16], [17]. CD8^+^ T cells recognize 8–10 amino acids long peptide epitopes in the context of MHC class I and upon activation, mount a specific cytotoxic response against virus-infected cells. In humans, a number of HLA-restricted RSV T cell epitopes have been found in the nucleocapsid (N) protein, F protein, M2-1 protein (encoded by the first of two open reading frames (ORFs) of the matrix protein (M2) gene), and the short hydrophobic (SH) protein [18], [19], [20], [21], [22]. In mice, epitopes specific for CD8^+^ T cells have been identified in F, G, matrix (M) and M2-1 proteins [23], [24], [25], [26], [27]. F protein elicits CTL responses in humans [19] and mice [24], [28] and enhances the production of Th1 cytokines, IL-2, IL-12 and IFN-γ. Therefore, identification of CD8^+^ T cell epitopes derived from F protein that are restricted to human HLA is important for the development of RSV vaccine.

The classical techniques used to enumerate CTL responses and epitope identification in humans has many limitations such as small blood sample volume that can be obtained safely from acutely infected young patients, the low frequency of RSV-specific memory CTL response in adults who were not recently infected [29], and the diversity of HLA class I gene polymorphism in donors. HLA-transgenic mice have been recognized as model to identity and study of epitope-specific CTL responses against viruses [30], [31], [32], [33], [34], [35]. In our study, we have predicted human HLA-A*0201-restricted CD8 epitopes from RSV F protein using the computational program named HLA Peptide Binding Predictions. These peptide epitopes have 9-mer amino acid core sequences and their association with the MHC class I molecule HLA-A*0201 was confirmed. These epitopes could also stimulate splenocytes from HLA-A*0201 transgenic (HLA-Tg) mice immunized with either RSV or recombinant adenovirus carrying F gene (rAd-F0) leading to enhanced production of IFN-γ and IL-2. In our system, peptide 13 (F214–222), peptide 14 (F273–281), and peptide 23 (F559–567) were found to be immunodominant, conferring viral clearance, and protection from RSV-induced body weight loss and lung pathology, with peptide 23 offering maximum protection. Collectively, the novel HLA-A*0201-restricted epitopes described here that elicit protective anti-viral responses are forerunners in the study of peptide-based RSV vaccine.

## Materials and Methods

### Ethics statement

All experiments were conducted in accordance with the guidelines of the Laboratory Animal Center of National Health Research Institutes (NHRI), Taiwan. The animal use protocols have been reviewed and approved by the NHRI Institutional Animal Care and Use Committee (Approved protocol no. NHRI-IACUC-098078-A).

### Animals and Cell lines

Eight to ten week-old female human HLA-A*0201 transgenic C57BL/6 mice, expressing cell surface HLA-A*0201, were gifted by Dr. Shih-Jen Liu, Institute of Infectious Disease and Vaccinology, NHRI, Taiwan, and C57BL/6 (H-2K^b^) and BALB/c (H-2K^d^) mice were purchased from National Laboratory Animal Center, Taiwan. The mice were maintained in pathogen-free conditions at the Animal Care Center of National Health Research Institutes throughout the period of study. Human T2 cells (purchased from the American Type Culture Collection, ATCC) (ATCC No. CRL-1992) expressing the HLA-A*0201 gene, but unable to present endogenous antigen, were cultured in IMDM (Hyclone)+10% fetal bovine serum (Biological Industries). Human embryonic kidney cells (293A) were purchased from Invitrogen (Cat. No. R70507). Human larynx carcinoma cells (HEp-2) were kindly obtained from Dr. Barney S. Graham, Vaccine Research Center, National Institute of Allergy and Infectious Diseases, National Institutes of Health, Bethesda, USA. The original HEp-2 cells were purchased from ATCC (ATCC No. CCL-23). 293A and HEp-2 were grown and maintained in DMEM medium (Hyclone) supplemented with 10% fetal bovine serum and 1% penicillin/streptomycin (P/S) (Biological Industries). All the cell lines were grown in an incubator maintained at 37°C and equilibrated with 5% CO_2_.

### Selection and synthesis of HLA-A*0201-restricted peptides

 Twenty-five HLA-A*0201- restricted 9-mer- peptide sequences (Table 1) were derived from the F protein of RSV-B1 strain using the computational program of HLA Peptide Binding Predictions developed by Bioinformatics and Molecular Analysis Section, National Institutes of Health (http://www-bimas.cit.nih.gov/molbio/hla_bind/). Epstein-Barr virus peptide, GLC-9 (GLCTLVAML) [36], known to be capable of binding to HLA-A*0201, was used as the positive control in T2 cell-stabilization assay. Hepatitis C virus capsid protein peptide “RPQPRGRRQPIPKARQPEGR” (HCV C55-74), which is a specific CD4 epitope for C57BL/6 mice, was used as the negative control. All the peptides were commercially synthesized by Echo Chemic, Ltd. and had 95% purity as confirmed by high-performance liquid chromatography. These peptides were dissolved in DMSO and diluted in the culture medium.

**Table 1:** Characteristics of HLA-A*0201-restricted epitopes of peptide spanning RSV F protein*.

No.	Sequence	T2-binding Ratio of MFI	IFN-γ ELISPOT		IL-2 ELISPOT	
			rAd-F0	RSV	rAd-F0	RSV
N	RPQPRGRRQPIPKARQPEGRL	1.00	N/a**	N/a	N/a	N/a
EBV	GLCTLVAML	1.96	N/a	N/a	N/a	N/a
1	TLLLWVLLL	1.19	33.5	69.5	85.5	39.5
2	LLWVLLLWV	0.97	46.5	46.5	34.5	42
3	AITTILAAV	1.71	68.5	119.5	9	31
4	ELDKYKNAV	0.94	88.5	34.5	49.5	51.5
5	FMNYTLNNT	0.96	73	12.5	38	58.5
6	FLLGVGSAI	1.14	65	61.5	29	32
7	HLEGEVNKI	0.83	68.5	81	68	33
8	ALLSTNKAV	1.50	36	86	8.5	43
9	LLSTNKAVV	1.53	27.5	1	5	2
10	STNKAVVSL	0.91	43	9.5	0	39.5
11	SLSNGVSVL	1.06	68.5	200	51.5	56.5
12	VLTSKVLDL	0.99	95.5	116.5	44	36
13	KVLDLKNYI	1.26	200	103.5	85	35.5
14	YMLTNSELL	1.71	200	97	200	34.5
15	KLMSNNVQI	1.51	39	0	37	59.5
16	LMSNNVQIV	1.60	54	28	52	50.5
17	KIMTSKTDV	0.95	24.5	2.5	24.5	6.5
18	SVGNTLYYV	1.29	115	92.5	0	42
19	KINQSLAFI	1.08	200	7.5	52	36.5
20	IMITTIIIV	1.03	200	9	32	24.5
21	IIIVIIVIL	0.88	16.5	80	2.5	21
22	VIIVILLSL	0.97	88.5	49	21.5	55
23	VILLSLIAV	1.51	130.5	87.5	34.5	43.5
24	LLSLIAVGL	1.71	84.5	0	5	42
25	SLIAVGLLL	1.42	41	96	31	44.5

**Figure 1:**
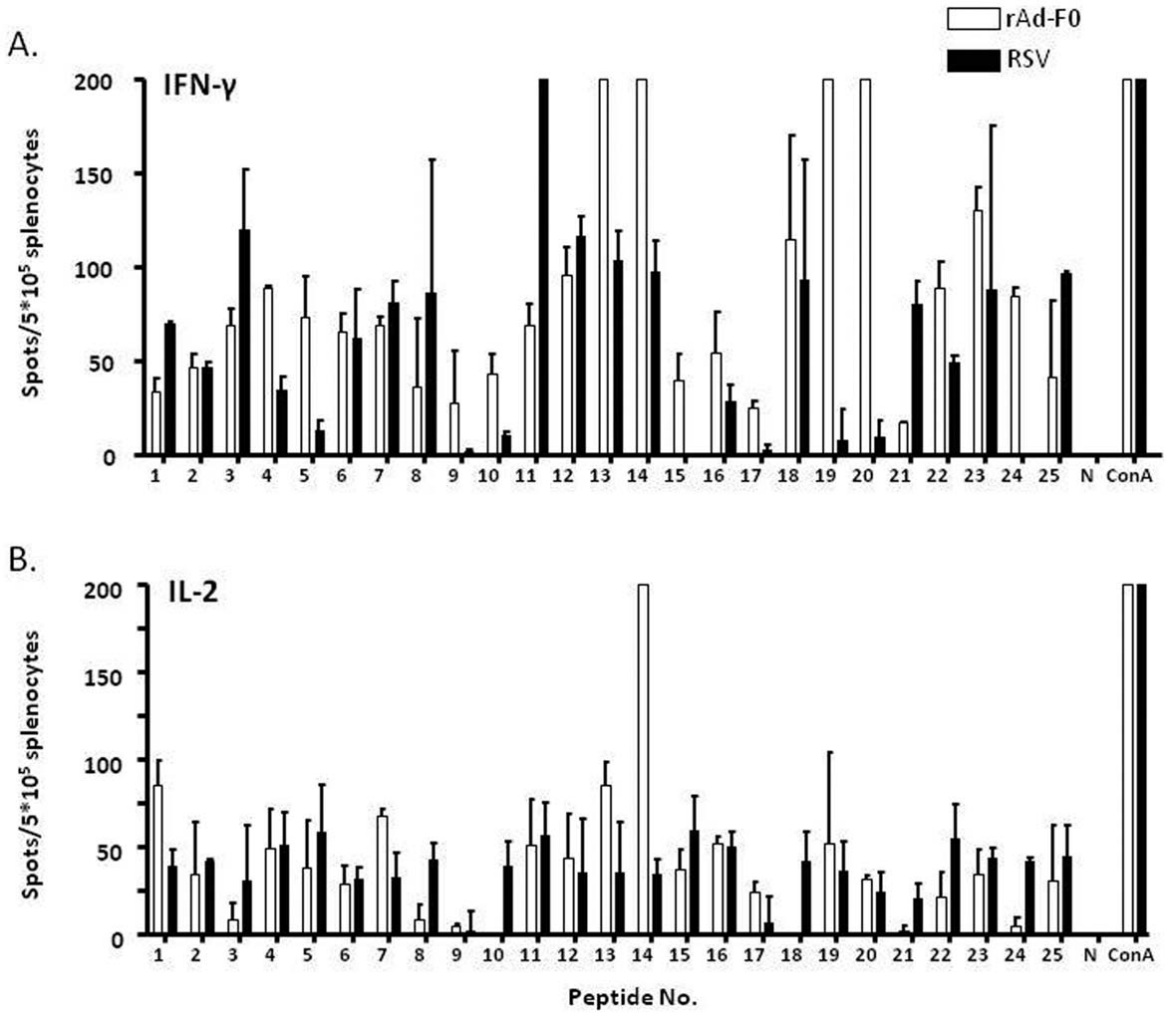
Induction of IFN-γ and IL-2 in splenocytes from RSV F peptide immunized HLA-A*0201 transgenic mice upon secondary recall. Splenocytes harvested on day 30 from HLA-A-Tg B6 mice primed and boosted at 20 days interval intranasally with 10^7^ pfu of rAd-F0 (□) or 10^4^ pfu of RSV-B1 (▪)were restimulated with 2 µg of the individual RSV F peptide or 10 µg/ml Con A for 5 days in the presence of murine IL-2 . After stimulation, 5×10^5^ splenocytes were seeded to anti-IFN-γ (A) or anti-IL-2 (B) capture antibody coated ELISPOT plates for 2 days for ELISPOT assay as described in Materials and Methods. Cytokine-positive immunospots were developed and the results are expressed as the number of immunospots +/−2 standard deviations for each experimental group. Data is representative of results derived from two independent experiments, each with five mice per group.

*Twenty-five HLA-A*0201-restricted 9-mer synthetic peptides from F protein of RSV-B1 strain were tested their binding ability with HLA-A*0201 by T2-stabilization assay, which has been described in the Materials and Methods. The study is a representative of results derived from two independent experiments, each with five mice per group. Results of IFN-γ and IL-2 ELISPOT assays have been described in the legend of figure 1.

**N/a, not assayed.

### T2 cell-stabilization assay

T2 cells (5×10^5^) cultured in 96-well U-bottom plates were incubated with the individual 9-mer synthetic peptides (50 µg/mL) and β2-microglobulin (5 µg/mL; Sigma-Aldrich) at 28°C for 16–18 hours. Subsequently, brefeldin A (10 µg/mL) was added and the cells were incubated at 37°C for another 3 hours, then washed and stained with fluorescence isothiocyanate (FITC)-conjugated mouse anti-human HLA-A2 antibody (SeroTec). Peptide-loaded T2 cells were acquired and analyzed on the FACScan flow cytometer using CellQuest software (Becton Dickinson). GLC-9 peptide was used as positive control (PC). The mean fluorescence intensity (MFI) represents the binding affinity of each peptide compared to MFI (set as MFI = 1) obtained from negative control (NC), which is T2 cells loaded with HCV peptide C55-74.

### Preparation of RSV-B1 strain stocks and rAd-F0

The propagation of human RSV-B1 strain VR-1580 (purchased from the American Type Culture Collection) in HEp-2 cells was described previously [37]. The virus was collected and resuspended in PBS, pH 7.2 and the titer was determined by a standard plaque assay. Briefly, 100 µL of varying dilutions of purified virus preparations was added to 5×10^5^ HEp-2 cells in a 12-well plate (Corning). Each culture was then overlaid with DMEM containing 1.5% methylcellulose (Sigma-Aldrich) and incubated for 5 to 6 days for the plaques to develop. Plaques stained with hematoxylin and eosin (H/E) were counted under a light microscope. The viral concentration is expressed as plaque-forming units per mL (pfu/mL). Propagation of rAd-F0 has been described previously [37]. Purification and concentration of the rAd-F0 was achieved by ultracentrifugation through a 15% sucrose/PBS gradient at 20,000 rpm for 60 min.

The virus was then resuspended in PBS, pH 7.2, and the titer determined by the modified plaque assay described above. Briefly, varying dilutions of rAd-F0 virus was added to 293A cells plated in a 12-well tissue culture plate. After overlaying the cultures with DMEM containing 0.75% methylcellulose, the cultures were incubated at 37°C for 10 to 12 days and plaques stained with H/E were counted. The yield of rAd-F0 was ∼1×10^9^ pfu/mL.

### Immunization and live RSV challenge of mice

To screen the immunodominance of epitopic peptide in the induction of RSV-F specific Th1/Th2 expression, HLA-A*0201 transgenic C57BL/6 mice were anesthetized with isoflurane and immunized with 1×10^7^ pfu/50 µL of rAd-F0 or rAd-LacZ or 1×10^4^ live RSV B1 *via* the intranasal (*i.n.*) route. Twenty days later, the mice were given booster immunization *i.n.* with the same dose. After 10 days of boost, mice were sacrificed and splenocytes were isolated for the cytokine ELISPOT assay. For peptide-based vaccination, transgenic mice were immunized subcutaneously (*s.c.*) with 50 µg of the individual synthetic peptides emulsified in 25 µL of incomplete Freund's adjuvant (IFA) (Sigma-Aldrich) and then boosted after 10 days *s.c.* with the same dose of the respective peptides mixed with IFA. Mice were sacrificed 7 days after the booster and splenocytes were isolated for *in vitro* peptide-restimulation. For challenge studies, 10^7^ pfu of live RSV-B1 was administered *i.n.* seven days after the booster immunization with peptide vaccines. Mice were sacrificed on day 4 post-challenge and the lung tissues were collected and subjected to further analysis by flow cytometry, ELISA, CTL assay and immunohistochemistry. Mice body weight was monitored daily.

### Enzyme-linked immunosorbent spot (ELISPOT) assay

5×10^6^ RBC-free splenocyte suspensions prepared from the individual mice were seeded in individual wells of 96-well filtration plates (Millipore) precoated with capturing monoclonal antibodies for murine IL-2, IL-4 or IFN-γ (0.5 µg/ well) (eBioscience) and blocked with conditioned medium (CM) for 1 hour at room temperature. The splenocytes were added with the individual RSV F 9-mer synthetic peptides (2 µg each) or GLC-9 dissolved in CM (100 µL). The splenocytes added with Con A (10 µg/mL) were used as positive control. Unstimulated splenocytes were used as negative control. The plates were kept in a 37°C incubator equilibrated with 5% CO_2_ for 48 hours. The individual wells of the ELISPOT plates were washed three times with washing buffer (0.05% tween20 in PBS, PBS-T), and then 0.2 µg of the corresponding biotinylated detection monoclonal antibodies specific for IL-2, IL-4 and IFN-γ were added to detect the respective cytokines. After 2 hours of incubation at room temperature, the plates were washed and 100 µl of streptavidin-alkaline phosphatase (1∶ 250 dilution) was added to the individual wells, and the plates were incubated at room temperature for 45 minutes. Finally, the plates were washed four times with the wash buffer, and 100 µl of AEC (3-amine-9-ethylcarbazole, Sigma-Aldrich) substrate was added to each well and allowed to react for 30 minutes at room temperature in the dark. The plates were then washed with water, air-dried overnight, and the spots per well were scored using the immunospot counting reader (C.T.L. IMMUNOSPOT, CELLULAR TECHNOLOGY LTD). Results were expressed as the number of cytokine-secreting cells per 5×10^5^ splenocytes seeded in the initial culture.

### In vitro peptide restimulation for T cell activation

The splenocytes were isolated, labeled with 5-(6)-carboxyfluorescein diacetate succinimidyl ester (CFSE) (Sigma-Aldrich) and stimulated *in vitro* with 10 µg/mL of the synthetic peptides for 4 or 8 days. Proliferation of splenocytic CD4^+^ and CD8^+^ T cells was analyzed by flow cytometry (BD FACSCalibur) using PE-Cy5-labeled specific antibody against CD4 (eBioscience) or CD8 (eBioscience), respectively. Cell division index (CDI), a ratio of CFSE-diluted CD4^+^ or CD8^+^ T cells stimulated by peptide to CFSE-diluted unstimulated CD4^+^ or CD8^+^ T cells, was calculated as shown below;
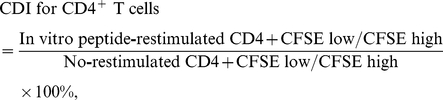


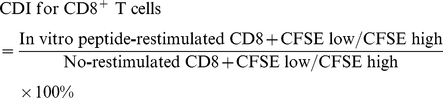
CDI represents the lymphocyte proliferation in response to individual peptide epitope stimulation.

For IFN-γ intracellular staining, splenocytes from peptide- or vehicle-immunized mice were stimulated *in vitro* with the respective peptides (peptide 3, 13, 14, and 23) (10 µg/mL) for five hours, washed with 1× PBS, and stained with anti-CD8 antibody conjugated with fluorescein isothiocyanate (FITC) (BD Biosciences) for 30 minutes, followed by subsequent fixation and permeabilization following the instructions of eBioscience fixation and permeabilization kit. The splenocytes were further stained for intracellular IFN-γ using PE-conjugated anti-IFN-γ antibody (BD Biosciences) for another 30 minutes. After washing, the samples were analyzed using flow cytometry.

### Preparation of lung homogenate

Whole lungs were perfused with 5 mL 1× PBS before excision. The isolated lung tissues were incubated with 1 µg/mL collagenase for 30 min before homogenizing with the iron mesh. The homogenate was centrifuged at 1,000 rpm for 10 min at 4°C to sediment the pulmonary cells. The cells were washed with 1× PBS and then resuspended in 1× PBS for further analysis by real-time RT-PCR and flow cytometry. For pulmonary IFN-γ detection, 5×10^6^/mL cells were cultured with 10 U/mL recombinant IL-2 for 2 days and the supernatants were collected for ELISA.

### Viral load determination

The PBS-perfused homogenized tissues were centrifuged at 1,000 rpm for 10 min at 4°C to sediment cell debris. Supernatants were collected, serially diluted, and tested for their ability to infect HEp-2 cells in the plaque formation assay as described above.

### Real-time RT-PCR

Lung RNA (5 µg) was mixed with 10 nmole random primers and 2 units of MMTV reverse transcriptase (Invitrogen) and incubated at 94°C for 10 minutes, 37°C for 30 minutes, and then at 4°C. The resulting cDNA was subjected to quantitative PCR analysis (The LightCycler® 480 Real-Time PCR system) with RSV N specific primer pairs, forward: 5′-aagatgcaaatcataaattcacagga-3′ and reverse: 5′-tgatatccagcatctttaagtatctttatagtg-3′. Expression of mouse GAPDH gene in the tested samples was detected as internal control by using specific primer pairs, forward: 5′-acccagaagactgtggatgg-3′ and reverse: 5′-acacattgggggtaggaaca-3′. The number of cycles (Ct) required for the amplification of the N gene and GAPDH was calculated. The relative expression of the N gene was calculated as followed: the individual Ct (RSV N) obtained from peptide- or vehicle-immunized lung was normalized by the respective Ct (GAPDH), and then 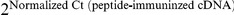 was divided by the mean of 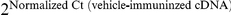. For detection of expression of chemokine genes in the lungs, primer pairs specific to CCL11, forward: 5′-tccacagcgcttctattcct-3′ and reverse: 5′-ctatggctttcagggtgcat-3′, to CCL17, forward: 5′-agtggagtgttccagggatg-3′ and reverse: 5′-ctggtcacaggccgttttat-3′, and to CCL22, forward: 5′-aaatgctcgccaatgatacc-3′ and reverse: 5′-aaggaagccaccaatgacac-3′ were used. To measure pulmonary cytokine expression in mice, primer pairs specific to IL-13, forward: 5′-cagctccctggttctctcac-3′ and reverse: 5′-ccacactccataccatgctg-3′, to IL-17a, forward: 5′-gattttcagcaaggaatgtgg-3′ and reverse: 5′-cattgtggagggcagacaat-3′, and to IL-18, forward: 5′-acgtgttccaggacacaaca-3′ and reverse: 5′-acaaaccctccccacctaac-3′ were used. The relative expression of the chemokine gene was calculated as the same as RSV N gene described above. All primer sets were synthesized commercially by Echo Chemic, Ltd, Taiwan.

### ELISA

The supernatants from 2-day culture of pulmonary cells were analyzed by calorimetric sandwich IFN-γ ELISA kit (eBioscience). Briefly, 96-well plates were coated with100 µL per well of anti-IFN-γ capturing monoclonal antibodies (1∶1000) in carbonate-coated buffer. The plates were incubated at 4°C overnight following by incubation with 1× assay diluent for one hour at room temperature. After three washings with PBS-T, biotinylated anti-mouse IFN-γ antibody (1∶1000) was added into each well for 30 minutes. The plate was washed with PBS-T and 100 µL of avidin conjugated with horseradish peroxidase (1∶250) was added to the individual wells for 30 minutes. The reaction was developed by 100 µL TMB substrate (3, 3′, 5, 5′-etramethyllbenzidine) for 20 minutes incubation in a dark room, and then terminated by adding 50 µL of 2 N H_2_SO_4_. The optical densities at 450 nm were determined with a microplate absorbance reader (SPECTRA, MAX2, M2).

### Isolation and culture of dendritic cells

Dendritic cells (DCs) isolated from HLA-A*0201-Tg B6 mice were used as target cells for CTL assay. Briefly, tibia were isolated from 6–8 weeks old mice and rinsed with 75% alcohol before their incubation with lymphocyte culture medium (LCM, RPMI containing 5% FBS, 20 mM HEPES, 50 µM 2-mecaptoenthanol, 1× P/S). The myeloid tissues were removed from the tibia with LCM and filtrated through 40 µm cell strainer (BD Falcon). The filtrates were centrifuged at 1200 rpm for 5 minutes and the cell pellet was resuspended in 2 mL RBC lysis solution (eBioscience), and incubated for 5 minutes followed by addition of 10 mL of 1× PBS buffer to stop lysis. Cells were centrifuged, washed once with 1× PBS, and resuspended in LCM containing 100 U/ml GM-CSF and cultured in an incubator maintained at 37°C, equilibrated with 5% CO_2_ for 6 days. At day 6, 10 µg/mL peptide epitopes and 5 µg/mL β2-microglobulin were added to the DC culture and incubated for another 24 hours. The peptide-pulsed DCs (10^6^) were treated with 50 ng/mL lipopolysaccharide (LPS) for one hour, labeled with CFSE in 1 mL of 1× CFSE staining solution, incubated at room temperature for 15 minutes, and then centrifuged at 400× g for 5 minutes to remove the supernatant, and were used as target cells in CTL assay.

### CTL assay

A non-radioactive assay for cell-mediated cytotoxicity using a green fluorescent probe CFSE to label target cells in combination with 7-amino-actinomycin D (7AAD), a red fluorescent probe to label dead effectors and target cells [38], was used to assess the presence of RSV F protein-specific CTLs in spleens or lungs of the immunized animals. The assay was carried out in accordance with the manufacturer's instructions (Cayman Chemical, Michigan, USA. catalog No. 600120). The assay entailed culturing splenocytes (5×10^6^) with a tested synthetic peptide (5 µg/mL) known to be a dominant HLA-A*0201-restricted CTL epitope in the presence of recombinant IL-2 (10 U/mL) at 37°C for 4 days. Peptide-loaded CFSE-labeled DCs isolated from HLA-Tg B6 mice were used as target cells. Un-pulsed DCs labeled with CFSE served as negative control. The target cells were resuspended in the culture medium and incubated at 37°C in a CO_2_ incubator for 30 minutes and then plated onto 12-well plates (10^4^/well, 250 µL) and co-cultivated with an appropriate number of *in vitro* restimulated splenocytes (as effectors) in defined effector∶target (E∶T) ratios in triplicates. Four hours later, the cell mixture was centrifuged and the pellet was resuspended in the 7AAD staining solution (1 mL), and kept in the dark for 15 minutes at 4°C. The cells were re-centrifuged again and the pellet was resuspended in 1 mL of assay buffer for analysis by flow cytometry. CFSE was measured in FL1 channel and 7-AAD in FL3 channel. The percentage of CFSE/7-AAD double positive target cells (i.e., DCs cell death) was analyzed. Target cells without CFSE or 7-AAD staining or only with CFSE staining were used as internal controls.

### Immunohistochemistry

Whole lungs were excised from the mice, fixed in 10% formalin (Sigma-Aldrich) solution overnight and embedded in paraffin (Thermo Fisher Scientific) for sectioning. The sections were stained with rat monoclonal anti-major basic protein (MBP) antibody (1∶500 dilution, gifted from Dr. James J. Lee, Mayo Clinic, AZ, USA) followed by the appropriate secondary antibodies. Twenty-bright field microscopy pictures (Nikon DXM1200 CCD digital camera attached with ACT-1 imaging capture software) from each stained section were taken at 200× magnifications and the number of eosinophils was counted. The mean of eosinophil count from each section was calculated.

### Statistical analysis

Unpaired, two-tailed student *t* test was used to compare the results obtained from the different experimental groups. Results are considered statistically significant when the p value is <0.05. The symbols * and ** are used to indicate p values<0.05 and <0.01, respectively.

## Results

### Screening of synthetic peptides for HLA-A2 binding and T lymphocyte activation

Twenty-five 9-mer synthetic peptides derived from the RSV F glycoprotein sequence presumed to encompass HLA-A*0201 binding motifs were synthesized (Table 1). Each was tested for its ability to bind HLA-A*0201 molecules by T2 cell-based binding assay (data not shown). The relative mean fluorescence intensity (MFI) obtained from the positive control (PC) peptide derived from Epstein-Barr virus capsid protein (GLC-9, a previously defined HLA-A*0201-restricted CTL epitope) was twofold (MFI = 1.96) compared to the baseline MFI = 1 obtained from the negative control (NC) treated with an unrelated peptide HCV C55-74 (a C57BL/6-specific CD4 epitope). The peptides 3, 9, 14, 15, 16, 23, and 24 showed 1.5 fold greater MFI than the negative control. The peptides 1, 6, 8, 13, 18, and 25 showed only modest binding affinity (∼10% higher than the baseline). The remaining peptides had no detectable binding to the HLA-A*0201 molecules. Apparently, the different MFIs generated by binding of RSV F-derived peptides to HLA-A*0201 reflects on the binding specificity of different peptides to a given HLA molecule.

To screen these peptides for their ability to activate RSV F-specific T cells, HLA-A*0201 transgenic C57BL/6 mice (HLA-Tg B6) were primed and boosted with rAd-F0- or RSV B1, splenocytes were harvested 10 days after the boost and then restimulated *in vitro* with the individual RSV F peptides. The peptide HCV C55-74 was not included because it had no binding to HLA-A*0201 molecules. The production of IFN-γ and IL-2 was measured by ELISPOT assay. Expression of HLA-A*0201 in the splenocytes isolated from HLA-Tg B6 mice was confirmed by flow cytometry using FITC-conjugated HLA-A*0201-specific antibody (data not shown). The peptides 3, 11, 12, 13, 14, 18, 19, 20, and 23 induced high levels of IFN-γ (over 100 spots) in the restimulated splenocytes. On the other hand, the peptides 1, 2, 4, 5, 6, 7, 8, 16, 21, 22, 24, and 25 only elicited moderate IFN-γ secretion (over 50 spots) (fig. 1A). Peptide 14 induced high production of IL-2 (over 100 spots), while the peptides, 1, 4, 5, 7, 11, 13, 15, 16, 19, and 22 induced moderate levels of IL-2 (over 50 spots) (fig. 1B). IL-4 production was minimal in all the samples (data not shown) indicating that Th1 rather than Th2 immune response was activated. Ultimately, among these CD8 epitope candidates, the critical factors to determine a suitable peptide are strong binding to HLA-A*0201 (ratio of MFI >1.2) and IFN-γ cytokine production (over 60 spots in both RSV and rAd-F0 immunization). Based on these criteria, the peptides 3 (F33–41), 13 (F214–222), 14 (F273–281), and 23 (F559–567) (Table 1) were chosen for further examination of the efficacy of peptide-based vaccination in HLA-Tg B6 mice.

### Evaluation of immunogenicity of the peptide epitopes in HLA-A*0201 transgenic B6 mice

**Figure 2:**
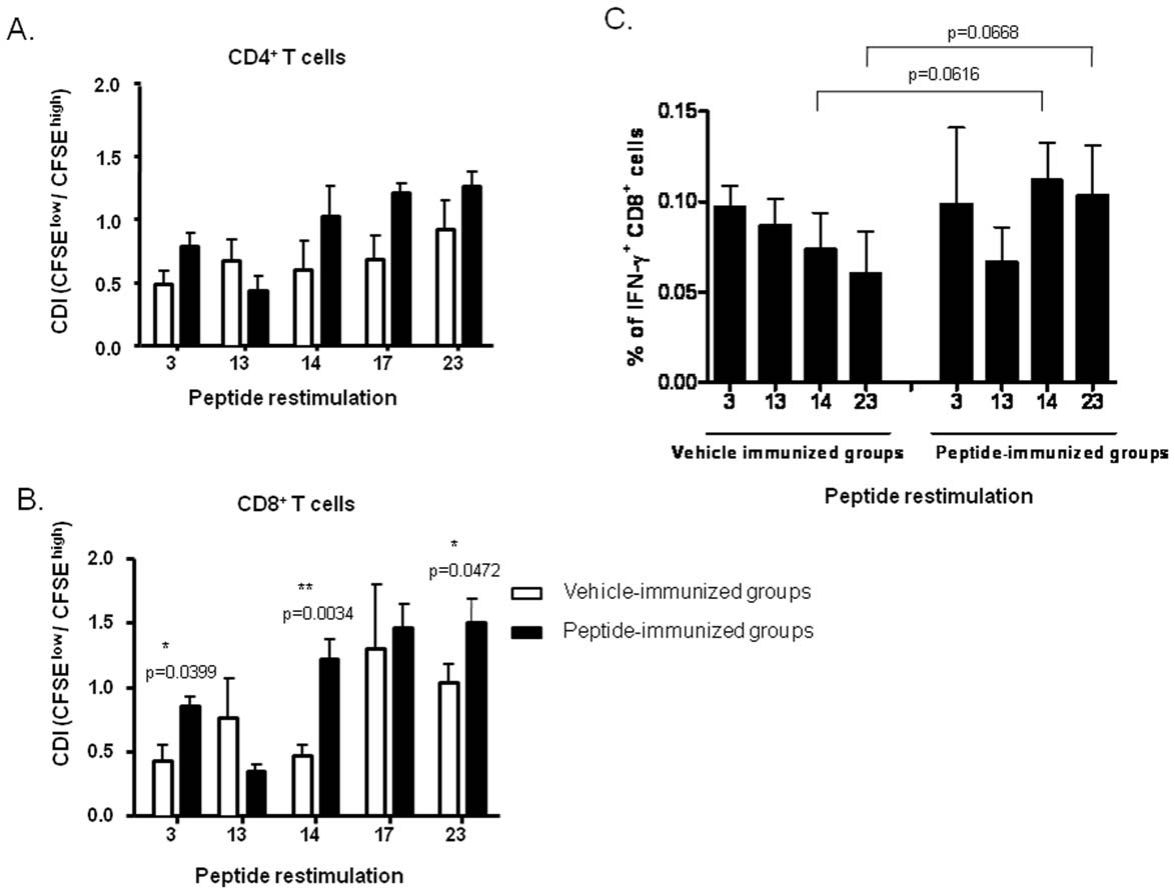
Epitope-specific CD4^+^ and CD8^+^ T-cell activation in peptide-immunized HLA-A*0201 transgenic mice. Splenocytes were isolated on day 17 from mice immunized twice subcutaneously with the peptides 3, 13, 14, 17, 23, or vehicle at day 0 and day 10. The splenocytes were labeled with CFSE and cultured in the presence or absence of 10 µg/mL of the respective peptides for 8 days. Proliferation of CD4^+^ (A) or CD8^+^ (B) lymphocytes in response to the different CD8 epitopes was analyzed by flow cytometry using anti-CD4 or CD8 antibodies conjugated with PE-Cy5. Results are presented as cell division index (CDI) as described in the Materials and Methods. (C) The splenocytes were stimulated *in vitro* with or without the peptides and were stained with anti-CD8 antibody conjugated with FITC, and then fixed and stained for intracellular IFN-γ using PE-conjugated anti-IFN-γ antibody. The percentage of CD8^+^ IFN-γ^+^ T cells was calculated. *(*p*<0.05) and **(*p*<0.01) indicate they are significantly different from the unstimulated splenocytes. Data is representative of results derived from three independent experiments.

To test the immunogenicity of the selected peptides, HLA-Tg B6 mice were immunized twice with the individual IFA-emulsified peptides 3, 13, 14, 17, or 23, at day 0 and day 10. Seven days post boosting, the splenocytes were harvested, labeled with CFSE, and restimulated with the respective peptides. In these experiments, peptide 17 with only a background binding to HLA-A*0201 and lower induction of Th1 cytokines (Table 1) was included as a control. Proliferation of CD4^+^ and CD8^+^ lymphocytes in response to peptide stimulation was analyzed by flow cytometry and the cell division index (CDI) was calculated as described in the Materials and Methods. Neither CD4^+^ nor CD8^+^ lymphocyte proliferation was detected 4 days after restimulation (data not shown). After 8 days of stimulation, it was observed that the peptides 3, 14, 17, and 23 induced insignificant proliferation of CD4^+^ T cells (Fig. 2A) while the proliferation of CD8^+^ T cells was ∼2-fold in response to peptide 3, ∼3-fold in response to peptide14, and ∼1.5-fold in response to peptide 23 (Fig. 2B). Based on these observations, peptide 17, which could not induce proliferation of CD8^+^ T cells, showed poor binding to HLA-A*0201 and lower induction of Th1 cytokines and was excluded from further investigations.

It has been reported that in BALB/c mice infected with RSV, activated IFN-γ^+^/CD8^+^ T cells generated against the viral proteins, F, matrix 2 (M2) and N played a protective role in viral clearance [23], [28], [39], [40]. We found that immunization with the peptides 14 and 23 marginally increased the proportion of IFN-γ^+^/CD8^+^ T lymphocytes due to some variation (Fig. 2C). We did not find any specific anti-epitope antibody in the sera from all groups of the mice (data not shown). In summary, only peptides 14 and 23 were found capable of activating CD8^+^ lymphocytes triggering proliferation as well as cytokine production.

### Efficacy of the CD8 epitope-based vaccine against live RSV

The immunoprotective ability of the individual CD8 epitopes derived from RSV F protein against live RSV challenge was assessed in peptide-immunized HLA-Tg B6 mice. The parameters selected to assess protection were the measurement of viremia in the lungs and the recovery from virus-induced body weight loss.

**Figure 3:**
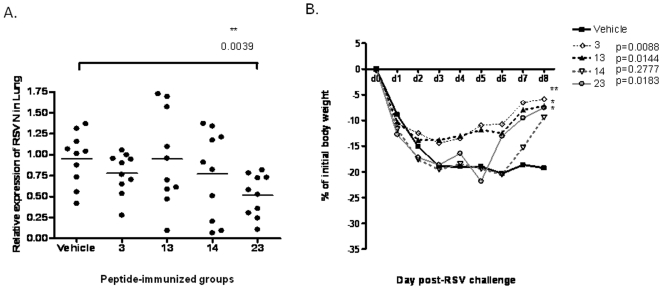
Determination of lung viral load and gain of lost body weight. Mice were immunized twice intranasally with vehicle (▪), peptide 3 (◊), peptide 13 (▴),peptide 14 (▽), or peptide 23 (□) before being intranasally challenged with 10^7^ pfu of live RSV B1. (A) The viral load in the lungs of individual mice was determined 4 days after challenge by real-time RT-PCR to quantitate RSV N gene expression as described in the Materials and Methods. 10 mice per group were used and the results are expressed as the relative expression of N gene normalized to GAPDH gene expression for each mouse. *(p<0.05) indicates they are significantly different from the vehicle-immunized group. (B) The body weight of each mouse was recorded daily for 9 days after virus challenge. Results are expressed as % (mean) for 5 mice in each experimental group. Two independent experiments were performed and data from one is shown. P value <0.05 calculated for peptide 3, peptide 13, peptide 14, and peptide 23 indicates they are significantly different compared to vehicle-immunized control.

The presence of viremia in the lungs of peptide-immunized mice challenged with RSV was measured. A more sensitive and accurate assay, real time RT-PCR specific for RSV N gene as described in the Materials and Methods was carried out, to determine the viral load in the lungs. The N gene encodes a RNAse-resistant nucleocapsid, which binds to genomic and antigenomic viral RNA in the process of viral particle formation [41]. Quantification of RSV N gene expression with real time RT-PCR to score clinical lung viremia has been shown to be a better method than plaque assay [42]. At the peak of virus replication (day 4 post-infection), a marked reduction in the expression of RSV N gene (mean = 0.51) was observed in mice immunized with two doses of peptide 23 followed by challenge with 1×10^7^ pfu of live RSV-B1 virus, as compared to animals administrated with the vehicle (mean = 0.95). Animals pre-immunized with the peptides 3, 13 or 14 showed no significant reduction of the viral load (Fig. 3A). Viremia was also noted to be undetectable on day 7 post-infection in animals immunized with each of these peptides (data not shown).

Primary RSV infection of adult BALB/c mice has been observed to cause weight loss in the early stages [11]. We monitored the body weight of mice immunized with the different peptides and observed loss of body weight upon RSV challenge in all the groups. Maximal body weight loss (about −20%) was observed two days post the viral challenge, following which, the mice were still unable to recover until day 8. Significant recovery of body weight loss was observed on day 6 to day 8 in peptide-immunized mice. Mice immunized with peptides 3, 13, or 23 showed better recovery of body weight (−11%, −12%, or −12%, respectively, on day 6) in the subsequent days compared to vehicle-immunized mice. Peptide 14 immunized mice recovered only in the last two days (Fig. 3B). However, the completely recovery of body weight loss by these CD8 peptide immunization is still limited.

### Proinflammatory cytokines induced by peptide immunization

Many studies have indicated a direct role for CD8^+^ T cells in mediating vaccine-enhanced disease [26], [27], [43], [44] and the chemokines CCL11, CCL17, and CCL22 are reported to be important in the development of RSV vaccine-enhanced pulmonary inflammation [16], [45], [46]. Lung cytokine profile of HLA-Tg B6 mice immunized with the different peptide epitopes (3, 13, 14 and 23) was analyzed 5 days after RSV challenge to understand the effect of cytokines on RSV F-specific CD8^+^ T cell response. Real-time RT-PCR analysis of RNA isolated from the lungs of the immunized mice was carried out using specific primers to detect CCL11, CCL17, CCL22, IL-13, IL-17, and IL-18. IL-13 is reported to be required for eosinophil entry into the lungs during respiratory syncytial virus vaccine-enhanced disease [47]. Pulmonary levels of CCL11 and CCL22 protein were significantly reduced in IL-13-deficient mice indicating that IL-13 mediates the recruitment of eosinophils into the lungs by inducing the production of Th2 chemokines and eosinophil chemotaxis [47]. IL-17 is a CD4^+^ T cell-derived cytokine that has been shown to stimulate airway responsiveness and mucus secretion during RSV infection [48], [49].

**Figure 4:**
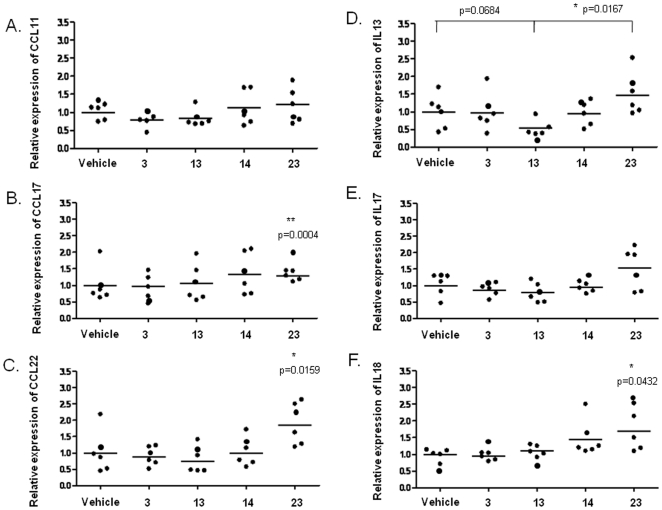
Expression of proinflammatory chemokines in the lungs of HLA-A*0201 transgenic mice immunized with CD8 epitopes and challenged with RSV. At day 4 post RSV challenge, lung RNA was extracted from the individual mice subcutaneously immunized twice with IFA-emulsified peptides or vehicle (IFA only). RNA was subjected to quantitative expression analysis of CCL11 (A), CCL17 (B), CCL22 (C), IL-13 (D), IL-17 (E), and IL-18 (F) by real-time RT-PCR using specific primers. GAPDH was used as internal control. The results are representative of the relative expression of the target gene normalized to GAPDH expression for the individual mouse. *(p<0.05) indicates the treatment is significantly different from the vehicle-immunized control. Similar results were obtained from two independent experiments, each with six mice per group, and one of them is shown.

We found that peptide 23-immunized mice showed significant upregulation of CCL17 and CCL22 (Fig. 4B and 4C), a marginal increase in IL-13 (Fig. 4D) but no upregulation of CCL11 or induction of IL-17 (Fig. 4A and 4E). The other peptides did not alter the induction of these chemokines. Peptide 13, on the other hand, decreased the expression of IL-13 in the lungs, compared to peptide 23 and vehicle (Fig. 4D).

IL-18 is a proinflammatory cytokine produced by macrophages, neutrophils, and airway epithelial cells that can enhance Th1 response [50], promote NK cell activation, and drive antiviral immunity in mouse infected with RSV coexpressing IL-18 [51]. We observed an increase in IL-18 expression in mice immunized with peptide 23 (Fig. 4F).

These results indicate that subcutaneous immunization of HLA-Tg B6 mice with peptide 23 followed by RSV challenge enhances the expression of inflammatory chemokines especially CCL17, CCL22, and anti-viral IL-18 in the lungs.

### Induction of pulmonary IFN-γ and CD8^+^ T cell function

Lung eosinophilia associated with RSV infection is known to cause bronchiolitis in children [52]. In murine studies, pulmonary illness and eosinophilia have been associated with immunization with FI-RSV vaccine as well as RSV G protein-encoding recombinant vaccinia virus (vacvG) [15], [53], [54], [55]. A study showed that RSV G protein was not an effector in the pulmonary eosinophilia and immunopathology induced by FI-RSV [56], indicating that the mechanism of RSV vaccine-enhanced disease is still poorly understood. This prompted us to investigate the safety aspect of the CD8 peptide-based vaccination by examining the correlation between vaccination and lung inflammation.

**Figure 5:**
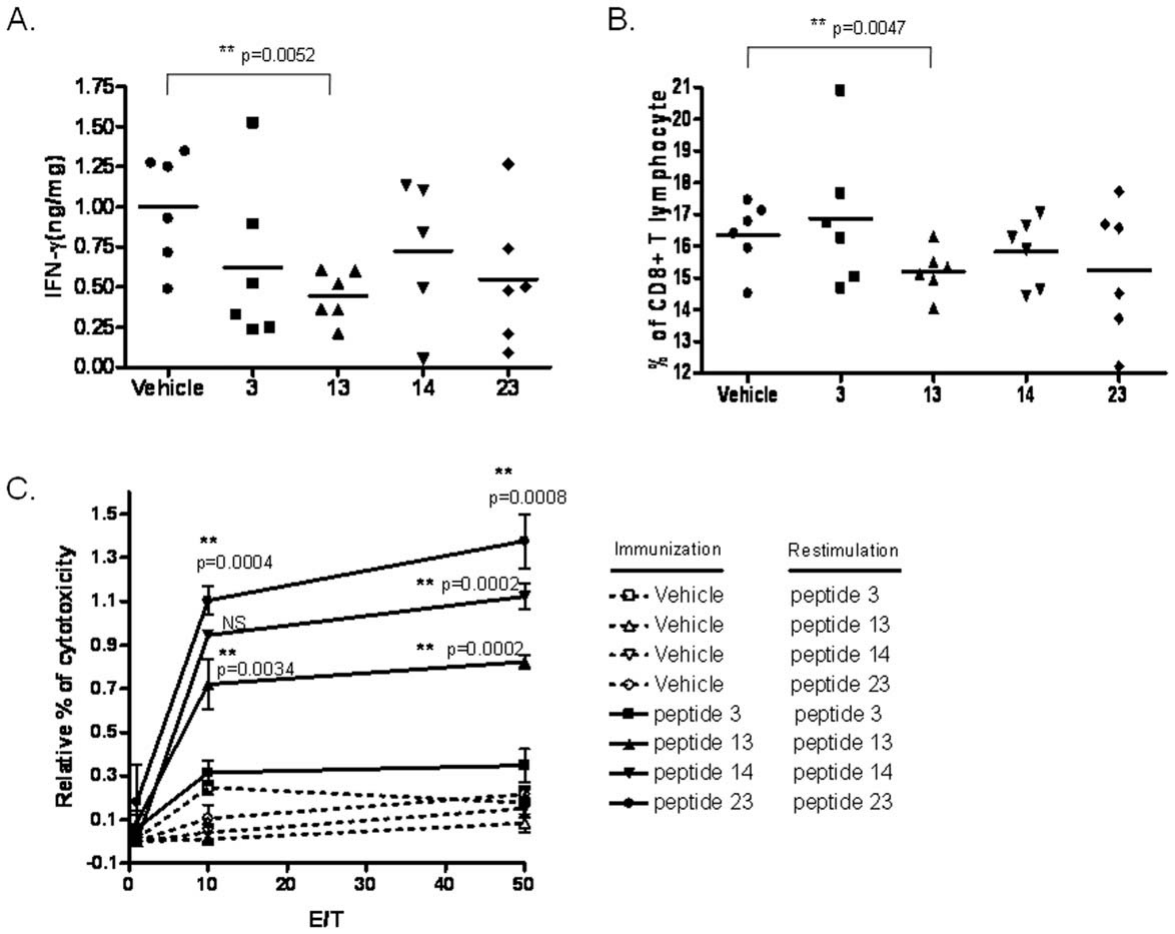
Induction of pulmonary IFN-γ and CTL activity in peptide immunized transgenic mice challenged with RSV. At day 4 post RSV infection, lung homogenates were prepared and the following were measured. (A) IFN-γ expression by ELISA, and (B) the number of CD8^+^ T cells in the lungs by flow cytometry using PE-cy5-labeled anti-CD8 antibody. (C). Enumeration of CTL activity in the lungs of mice immunized with CD8 peptide epitopes and challenged with RSV. Effector lymphocytes isolated from the lungs of mice immunized with peptide 3 (▪), peptide 13 (▴), peptide 14 (▾), or peptide 23 (•) were cultured and supplemented with murine IL-2 in the presence of 2 µg of the same peptide for 5 days. In parallel, cultured pulmonary lymphocytes from vehicle-immunized mice were stimulated with 2 µg of peptide 3 (□), peptide 13 (Δ), peptide 14 (▽), or peptide 23 (ο), respectively, for 4 days. DCs isolated from the tibia of HLA-B6 mice were pulsed with 20 µg per mL of the individual peptides for 2 hours at 37°C and labeled with CFSE and used as targets in the *in vitro* CTL assay. Un-pulsed DCs served as negative control. The viable effector cells were co-cultured with 10^4^ peptide-loaded target DCs cells at effector∶target ratios of 50∶1, 10∶1, and 0∶1 for five hours. The cell mixtures were labeled with 7-AAD and analyzed by flow cytometry. Results are expressed as mean percentage of 7-AAD/CFSE positive cells normalized with un-pulsed DCs. Six mice were taken in each group. The result is a representative of two independent experiments.

Transgenic mice immunized twice with the peptides were challenged with RSV and the secretion of IFN-γ in the lungs was measured. Four days after viral challenge, decreased IFN-γ secretion was found in mice immunized with the peptides, compared to vehicle (Fig. 5A). We further investigated whether the reduced expression of IFN-γ in peptide-immunized mice lungs was due to fewer number of CD8^+^ T lymphocytes infiltrated into the lungs. We found that peptide immunization did not lead to a profound increase in infiltrated pulmonary CD8^+^ T lymphocytes upon RSV challenge (Fig. 5B); in fact, CD8^+^ T cell numbers were lower when immunized with the peptides 13, 14, or 23, compared to vehicle immunization. But, the CTLs induced by the peptides 13, 14, and 23 were found to be efficient in target cell lysis when assessed by *in vitro* CTL assay using congenic HLA-Tg B6 mouse's dendritic cells as targets. The target cells were labeled with CFSE/7AAD (target) and loaded with the respective peptides and mixed with effector cells at the effector/target (E∶T) ratios of 10∶1 and 50∶1. Peptide 23 induced highly efficient CTLs with maximum lysis followed by peptides 14 and 13 (Fig. 5C). These results confirmed that peptides induced pulmonary CTLs activity but modulated the number of CD8^+^ T cells recruited to the lung.

### Pulmonary inflammation in immunized mice upon RSV challenge

**Figure 6:**
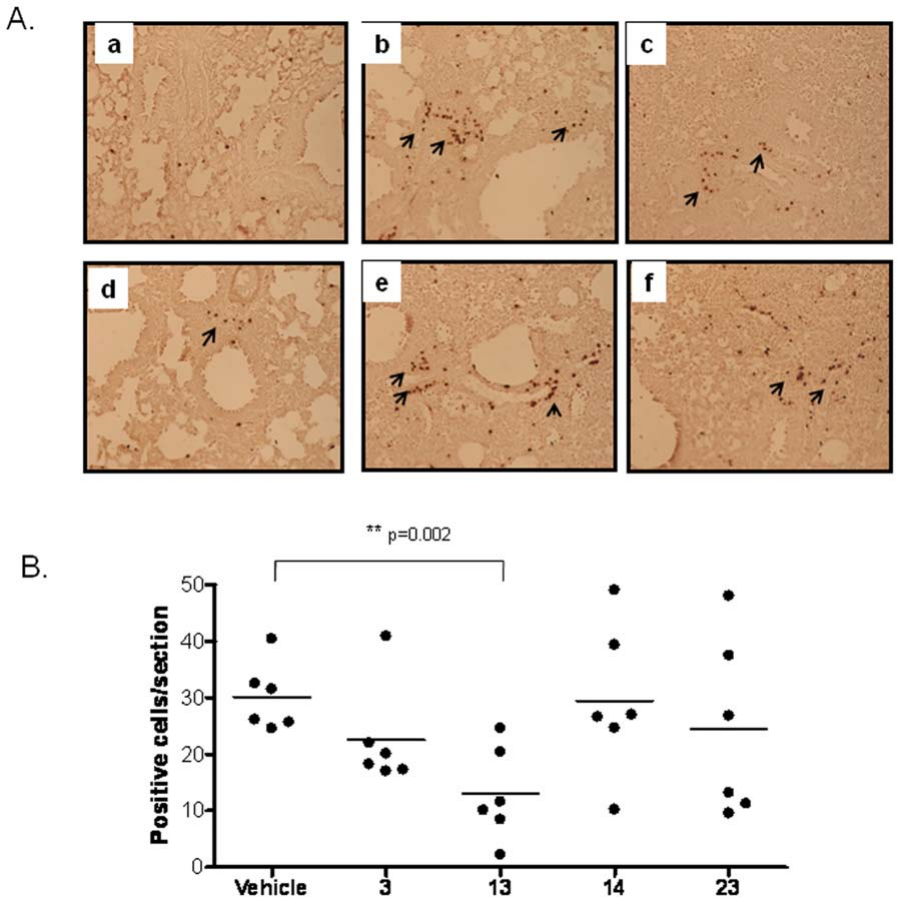
Eosinophil infiltration into the lungs of peptide-immunized transgenic mice upon RSV challenge. Immunohistochemical analysis of lung sections with anti-major basic protein antibody specific for eosinophils followed by the HRP-conjugated anti-rat antibody was performed. (A) Pictures from the section of normal mouse lung (a), or vehicle- (b), peptide 3- (c), 13- (d), 14- (e) and 23- (f) immunized mouse lung section. (B) Quantitative representation of eosinophil count per section of peptide- or vehicle-immunized HLA-transgenic mice at day 4 post RSV challenge. Twenty bright field pictures from each lung mesenchymal region were examined and the number of eosinophils was counted under 200× magnification. The mean number of eosinophils in each group, six mice per group, is represented. Similar results from two independent experiments were obtained and one of the results is shown.

To confirm whether CD8 epitope immunization followed by RSV infection in HLA-Tg mice can induce eosinophilia, we sectioned the lung tissues and stained with anti-major basic protein (MBP), which is a marker of eosinophilia. In the control HLA-Tg mouse lung, very few eosinophils were present in the lung mesenchyme (Fig. 6A). Primary RSV infection induced some clusters of infiltrated eosinophils in the mesenchymal tissues [57], as seen in the vehicle immunized mice infected with RSV. Peptides 3, 14, and 23 showed comparable eosinophil numbers in the lungs similar to vehicle-immunized mice (Fig. 6A). Eosinophil numbers were significantly reduced in the lungs of peptide 13-immunized mice (Fig. 6B). Indeed, prevention of pulmonary eosinophilia by peptide 13 also correlates to the lower secretion of IL-13 (an eosinophil chemotaxisic factor [47]) as shown in Fig. 4D. In summary, these results implicate the differential activity of HLA-A*0201-restriced CD8^+^ epitopes derived from RSV F. The peptides not only contribute to viral clearance (peptide 23, F559–567), but also reduce lung inflammation induced by RSV infection (peptide 13, F559–567) in HLA-Tg B6 mice.

## Discussion

CD8^+^ T cell response is crucial for the development of host adaptive immunity against viral infection. Previous studies conducted in mice [58], [59] and in human [18] showed that the RSV F protein elicited CTL responses conferred protection against live RSV infection. The CTL epitopes restricted to the murine K^d^ molecules have been identified to comprise of the amino acids F85–93, F92–106, and F249–258 of the F protein [24], [25], [60]. The residues F118–126, F551–559, and F109–118 have been found to be epitopes presented in the context of HLA-B*57, HLA-Cw*12, and HLA-A*01 alleles [19], [61]. Our studies show that the F protein of RSV is rich in motifs that bind to HLA-A*0201 molecules. Peptide 13 (F214–222), 14 (F273–281), and 23 (F559–567) presented by HLA-A*0201 as 9-mer, have been previously identified algorithmically [62], in which the immunogenicity of these epitopes was still unclear. The sequence of peptide 14 (YMLTNSELL) is covered by an identified mouse K^d^-restricted epitope, a 10-mer F249–258 (TYMLTNSELL) peptide [24], [25], [60], meant to be a cross-species of CD8 epitope located in the F protein of RSV.

The algorithmically predicted RSV F-specific CD8^+^ epitopes used in our study are confirmed to bind to human HLA-A*0201 as well as elicit Th1 cytokine response in HLA Tg mice infected with RSV or rAd-F0 (Table 1). Two of the synthesized peptides, 14 and 23, were found to be ideal as evident by their ability to activate CD8^+^ T cells (Fig. 2) in HLA-Tg mice. A direct correlation was observed between the magnitude of the CTL responses that were induced by these peptides and viral clearance. In HLA Tg mice challenged with live RSV, the maximum CD8^+^ activity was observed in peptide 23 immunizated mice (Fig. 5) along with enhanced secretion of inflammatory chemokines and NK activators such as CCL 17, CCL22, and IL-18 in the lungs (Fig. 4). These factors would have contributed to the observed enhanced viral clearance. Peptide 14 produced moderately efficient CTLs and therefore reduced lung viral load only marginally (Fig. 3A).

Interestingly, peptide 3 and 13 showed significant prevention of mice weight loss induced by live RSV infection (Fig. 3B) in spite of reduced activity of the elicited CTLs, as compared to peptide 14 or 23 that elicited stronger CTL activities in mice (Fig. 3C), where recovery of body weight loss was only mild (Fig. 3B). These results corroborate a previous report that CD8^+^ T cells induced in mice immunized with vacvF and challenged with live RSV contributed to body weight loss in the presence of Th1 cytokines [44]. The exact mechanism of prevention of RSV-induced weight loss by the peptides 3 and 13 derived from RSV F is still being investigated.

A recent study has generally questioned the role of CTLs in the pathogenesis of human RSV infection [63]. Very few CD8^+^ T cells were found in the lung infiltrates of human infants infected with lethal RSV. Additionally, the presence of T cell cytokines in the nasal secretion was also low and few CTLs were recovered from BAL of infants with bronchiolitis [63]. A similar phenomenon was observed in our study; it was noted that in HLA-Tg B6 mice immunized with IFA (vehicle) and infected with RSV, few pulmonary CD8^+^ T cells were recovered (Fig. 5), and the expression of inflammatory chemokines was low (Fig. 4). It is reported that a number of chemokines such as CCL2, CCL5, CXCL10 are associated with RSV-induced airway hyperresponsiveness [64], [65] and CCL11, CCL17, and CCL 22 are involved in the development of pulmonary eosinophilia with RSV infection [7], [16], [45], [46]. In murine model, development of pulmonary eosinophilia in BALB/c mice immunized with vacvG followed by RSV infection could be diminished by co-immunization with vacvG+vacvM2 that increased the number of M2_82_-specific CD8+ T cells and inhibited CCL17 and CCL22 expression in the lungs [7]. In our study, the cytokine profile of peptide 23-immunized mice depicting elevated CCL17, CCL22, and IL-18 might have contributed to the antiviral responses rather than the induction of lung inflammation, in which no significant eosinophilia was observed (Fig. 6).

Although a previous report showed that CD4^+^ CD25^+^ Foxp3^+^ Treg cells might be involved in enhancing the migration of virus-specific CD8 T cells into the lungs [66], we do not observe this phenomenon in our study. Instead, our unshown results show increased infiltration of CD4^+^CD25^+^ T cells in the lungs of mice pre-immunized with peptides 13, 14, or 23, and challenged with live RSV. This correlates with the reduced IFN-γ level and reduced infiltration of CD8^+^ T cells with normal CTL activity (Fig. 5).

Significant reduction of eosinophil counts in the lungs of mice immunized with peptide 13 (Fig. 6) correlates with the reduced number of infiltrated CD8^+^ T cells in the lungs (Fig. 5). Importantly, we also observed the level of IL-13 associated with the induction of eosinophilia in FI-RSV or vacvG-immunized mice challenged with live RSV [47], [67] was reduced (Fig. 4). Compared to vehicle-pre-immunized mice, pre-immunization with peptides 14 and 23 elicited normal expression of IL-13 (Fig. 4) and there was no enhanced recruitment of eosinophils to the lungs (Fig. 6). These results not only elucidate that these peptides derived from RSV F are able to prevent RSV-induced lung illness (eosinophilia) but also corroborate earlier reports that co-immunization with vacvF and vacvG individually inhibits vacvG-enhanced pulmonary eosinophilia upon RSV infection [14], [15], [68], [69], [70]. We believe that our study is the first to demonstrate RSV F-derived CD8 epitopes in the regulation of the lung inflammation induced by RSV infection.

Immunization with a single CTL epitope has previously been shown to induce protection against viral infection or tumor growth [71], [72]. In some cases, protective responses were only induced following immunization with CTL epitope linked to a T helper epitope or other carrier [73]. Our unpublished data shows that HLA-Tg B6 administered with two doses of peptide 23 mixed with HCV T helper epitope prior to challenge with RSV could not see the enhancement of antiviral activity in the lungs.

In conclusion, we have identified two distinct HLA-A*0201-restriced CD8 epitopes, peptide 14 (F273–281) and peptide 23 (F559–567) from RSV F that elicit dominant activation of CD8^+^ T lymphocytes, and are capable of moderate to significant viral clearance, and also help restoration of body weight loss induced by RSV infection. Other identified epitopes also elicit different anti-viral responses; peptide 3 (F33–41) and peptide 13 (F214–222) are capable of limiting body weight loss, and peptide 13 can prevent RSV-induced lung eosinophilia. These observations would allow us to further pursue the development of multiple-CD8 epitopes-based prototype RSV vaccine, which would not contribute to vaccine-enhanced diseases.
